# A Fluorescent Probe to Detect Quick Disulfide Reductase Activity in Bacteria

**DOI:** 10.3390/antiox11020377

**Published:** 2022-02-13

**Authors:** Ying Zhao, Xin Zuo, Shuang Liu, Wenjun Qian, Xuewen Tang, Jun Lu

**Affiliations:** Key Laboratory of Luminescence Analysis and Molecular Sensing, Ministry of Education (Southwest University), College of Pharmaceutical Sciences, Southwest University, Chongqing 400715, China; zhaoying_totoro@163.com (Y.Z.); heidi202201@163.com (X.Z.); liushuang_2022@163.com (S.L.); qianwj18@email.swu.edu.cn (W.Q.); 18832819339@163.com (X.T.)

**Keywords:** thioredoxin, glutathione, redox, bacteria, probe, fast disulfide reduction

## Abstract

The Trx and Grx systems, two disulfide reductase systems, play critical roles in various cell activities. There are great differences between the thiol redox systems in prokaryotes and mammals. Though fluorescent probes have been widely used to detect these systems in mammalian cells. Very few methods are available to detect rapid changes in the redox systems of prokaryotes. Here we investigated whether Fast-TRFS, a disulfide-containing fluorescent probe utilized in analysis of mammalian thioredoxin reductase, could be used to detect cellular disulfide reducibility in bacteria. Fast-TRFS exhibited good substrate qualities for both bacterial thioredoxin and GSH-glutaredoxin systems in vitro, with Trx system having higher reaction rate. Moreover, the Fast-TRFS was used to detect the disulfide reductase activity in various bacteria and redox-related gene null *E. coli*. Some glutaredoxin-deficient bacteria had stronger fast disulfide reducibility. The Trx system was shown to be the predominant disulfide reductase for fast disulfide reduction rather than the Grx system. These results demonstrated that Fast-TRFS is a viable probe to detect thiol-dependent disulfide reductases in bacteria. It also indicated that cellular disulfide reduction could be classified into fast and slow reaction, which are predominantly catalyzed by *E. coli* Trx and Grx system, respectively.

## 1. Introduction

Oxidative stress is one of the most common and challenging conditions for bacterial homeostasis and survival [1,2]. Prokaryotic microorganisms evolved various antioxidant systems to mediate the challenge [3,4]. Thiol-dependent redox systems play a fundamental role in maintaining the redox balance in bacteria [5,6]. Most Gram-negative bacteria have two primary thiol-dependent antioxidant pathways called thioredoxin (Trx) and glutaredoxin (Grx) systems that use nicotinamide adenine dinucleotide phosphate (NADPH) to reduce intracellular disulfides [6,7,8]. In the Trx system, electrons are transferred from NADPH to thioredoxin reductase (TrxR), then to Trx, while in the Grx system, it is glutathione reductase (GR) and glutathione (GSH), finally to glutaredoxins. *E. coli* has two Trxs including Trx1 and Trx2 encoded by *trxA* and *trxC*, and three glutaredoxins, Grx1, Grx2 and Grx3 encoded by *grxA*, *grxB*, and *grxC*, respectively [7]. Moreover, there are three transcriptional factors including OxyR, SoxR and SoxS in bacteria that play a key role in regulating the gene expression level of redox systems against oxidative stress [4,9,10]. OxyR also serves as a master regulator of S-nitrosylation [11].

Besides disulfide reductases, low-molecular-weight (LMW) thiols play a crucial role in maintaining the reduced state of protein thiols in cytoplasm in all organisms [12,13]. Glutathione (GSH) usually works as the most abundant LMW thiol of Gram-negative bacteria [14]. It is oxidized to GSSG under oxidative stress and its autooxidation rate is seven times slower than that of free cysteine [15]. GSH functions to protect bacterial cells from redox active compounds, antibiotics and toxic metals [16]. In addition, Cys residue could be a small compelling nucleophile because of its thiol (-SH) functional group [4,17]. Recent research shows *E. coli* could use Cys-SSS-Cys to generate Cys-SSH, which could protect cellular thiols from reacting with the electrophiles [18]. Gram-positive bacteria, such as *B. subtilis* and *S. aureus*, have bacillithiol (BSH) which may play a predominant role in the cytosolic thiol redox chemistry and it is functionally analogous to GSH [19,20].

In the last two decades, there has been a growing trend towards using fluorescent probes to determine the reactive sulfur species in vivo [21,22]. In recent years, studies have provided important information on the use of fluorescent probes to detect LMW thiols e.g., cysteine [23], as well as antioxidant enzymes, including mammalian TrxR [24] and Trx [25,26], whereas, there is a very limited understanding of cellular redox homeostasis in bacteria [27]. Besides the differences in the cell wall/cell membrane between bacterial and mammalian cells, there are great differences in the composition and function of thiol-dependent systems between two types of organisms. For example, the structure and activities of mammalian TrxR is different from bacterial TrxR [6]. Thus, the probe which can be used to detect mammalian TrxR activity, may not be suitable for the analysis of bacterial TrxR. In addition, mammalian cells and some Gram-negative bacteria have both Trx and GSH-Grx systems, while most Gram-positive bacteria including *B. subtilis* and *S. aureus* only contain Trx systems [6].

Trx system and Grx system have some specific substrates. Trx, not Grx, provides the electrons to thioredoxin-dependent peroxidase (peroxiredoxin), which scavenges reactive oxygen species quickly to maintain the redox balance and regulate redox signaling, whereas both Trx and Grx systems belong to the thioredoxin superfamily and share a similar structure with different electron donor [28,29,30]. Thus, they can transfer electrons to ribonucleotide reductases (RNR), which is essential for DNA synthesis and repair. However, the contribution of Trx and Grx in the disulfide reduction in vivo are not well investigated and clarified. The activity of *E. coli* Trxs is normally determined by catalyzing the reduction of insulin disulfides in vitro [31,32], and Grxs was measured by HED method [33]. Isothiocyanate-labeled insulin (FiTC–insulin) and eosin-glutathionylated BSA have been developed to detect Trx and Grx activity, respectively [34,35]. However, these methods are frequently used to measure Trx or Grx activity in tissue or cell lysates. Very few methods have been developed to analyze in vivo disulfide reductase activities, particularly in bacteria.

Recently, we have found the disulfide-containing probe TRFS-green is a common substrate for *E. coli* Trx and Grx systems. Therefore, we used TRFS-green to distinguish the contribution of Trx and Grx systems to redox regulation [36]. To our surprise, Trx system had higher reactivity towards TRFS-green than Grx system in vitro. However, *E. coli* Grx systems, particularly grx2 and grx3 contribute more to the reduction of TRFS-green compared to the thioredoxin system. This is agreeable with the early results from ELISA analysis that show grx2 and grx3 are the most abundant redox proteins in *E. coli* [7]. The limitation of this method is that the measurement needs to be monitored for 2 h, indicating that the disulfide reaction detected by the probe is a slow process, which is not suitable for real-time monitoring of fast in vivo disulfide reactions. Some bacteria such as *E. coli* have a quite fast growth rate, and a quick response to oxidative stress [3], which may require the fast disulfide reduction reaction to keep the redox balance.

The aim of this study is to explore in vivo detection of thiol-dependent regulation in bacteria. Particularly, we examined whether a fluorescent probe Fast-TRFS can be used to detect disulfide reductase in bacteria. Fast-TRFS is a disulfide containing fluorescent probe, which yields fluorescence only when its disulfide bond is reduced to dithiols. It has been used in a fast detection of mammalian TrxR activity. Thus, it is interesting to know whether the Trx system and Grx/GSH system activity in bacterial live cells can be detected by the probe to reveal changes in bacterial cellular redox state.

## 2. Materials and Methods

### 2.1. Microorganisms and Reagents

**Scheme 1:**
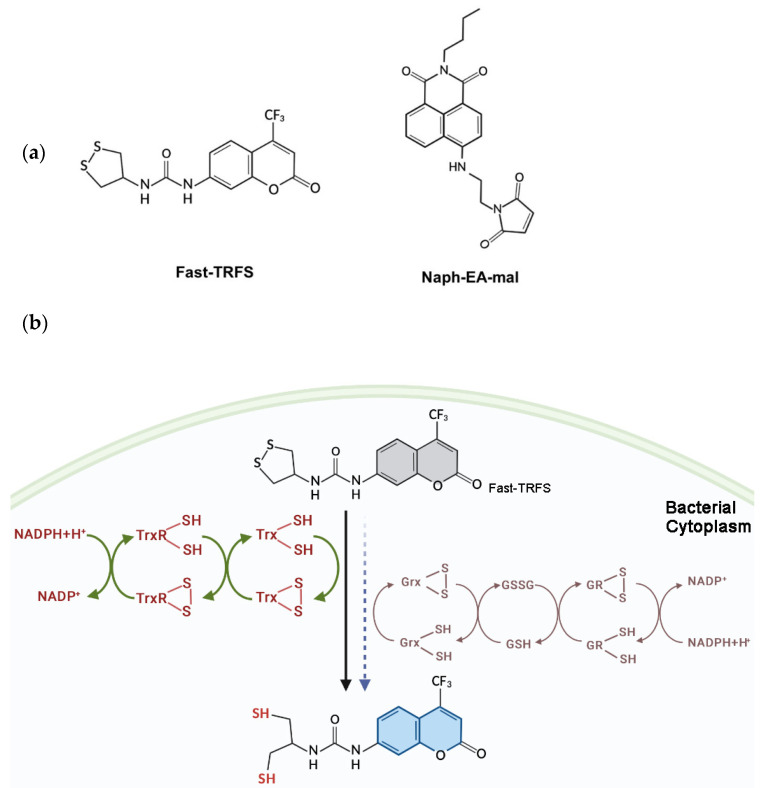
Structure of Fast-TRFS and Naph-EA-mal (**a**) and reduction of Fast-TRFS by bacterial Trx and Grx systems to yield blue fluorescence (**b**). Trx, not Grx, is the major player in catalyzing disulfide reduction with a fast reaction rate in *E. coli*.

Fast-TRFS and Naph-EA-mal were obtained from MedChemExpress, New Jersey, USA (www.medchemexpress.com, accessed on 20 January 2022). Their structures are shown in Scheme 1a. *Escherichia coli* DHB4 (*E. coli* DHB4, wild type) and *E. coli* mutants *trxABC*^−^ (Trx1, TrxR and Trx2 deficient), *trxA*^−^*grxA*^−^ (Trx1 and Grx1 deficient), *gshA*^−^ (GSH deficient), *gor*^−^ (GR deficient) and *grxABC*^−^ (Grx system deficient) were kindly provided by the Arne Holmgren laboratory at Karolinska Institutet, Sweden and described as before [7]. *E. coli* TrxR, Trx, and Grx were obtained from IMCO Corporation Stockholm, Sweden. Yeast GR, reduced GSH, Dithiothreitol (DTT) and NADPH were purchased from Sigma-Aldrich (St. Louis, MO, USA). *S. aureus* ATCC29213, *B. subtilis* ATCC14990, *B. cereus* ATCC14579, *P. aeruginosa* were from the American Type Culture Collection.

### 2.2. Reduction of Fast-TRFS by Thiol-Dependent Redox System In Vitro

Reduction of Fast-TRFS by the combination of various *E. coli* Trx and Grx system components in vitro was performed in 96-well panel. In brief, 10 µM Fast-TRFS was reacted with NADPH (200 µM), *E. coli* TrxR (50 nM), *E. coli* Trx (1 µM), yeast GR (50 nM), GSH (1 mM) and *E. coli* Grx (1 µM) in Tris (50 mM)/EDTA (1 mM) buffer (TE buffer, pH = 7.4). Fluorescent intensity (FI) was measured every 1 min over 15 min using a VERSA microplate reader at 37 °C, with excitation at 343 nm and emission at 451 nm. In addition, to test the effects of different Trx concentrations on the reduction of the probe, Fast-TRFS was incubated with NADPH (200 μM), TrxR (50 nM) and serial concentrations of Trx (0, 0.125, 0.25, 0.5, 1 µM). Likewise, to detect the effects of GSH concentrations on probe reduction, Fast-TRFS was incubated with NADPH (200 μM), GR (50 nM), Grx (1 μM), and serial concentrations of GSH (0, 0.125, 0.25, 0.5, 1 mM).

### 2.3. Observation of Fluorescence in E. coli with Confocal Microscopy

Observation of the fluorescent probes Fast-TRFS and Naph-EA-mal in bacterial cells was performed using a Nikon A1R+ laser scanning confocal microscope (Nikon, Japan). To detect Fast-TRFS, mid-log phase *E. coli bacteria* were treated with the probe for 5 min. Then, bacterial cells, at concentrations of 10^6^ colony forming units (CFU)/mL, were transferred to slides for observation under the confocal microscope. To detect total thiol level in bacteria, mid-log phase *E. coli* incubated with Naph-EA-mal the probe for 10 min were stained with 4′,6-diamidino-2-phenylindole (DAPI) (2.5 μg/mL) for 15 min at room temperature in the dark. Subsequently, 10^6^ CFU/mL *E. coli* were transferred to slide for analysis. The images were captured using 100× oil immersion. Excitation wavelength was 405 nm for Fast-TRFS stain and DAPI, 488 nm for Naph-EA-mal. Bright field images were also captured using transmitted light.

### 2.4. Detection of Thiol-Dependent Redox System in Bacteria

Different bacteria were used to compare the disulfide reducibility and total thiol level through Fast-TRFS and Naph-EA-mal probes. *S. aureus* ATCC29213, *B. cereus* ATCC14579, *P. aeruginosa*, *B. subtilis* ATCC14990 and *E. coli* DHB4 wild type were cultured overnight and then diluted 1:100 to grow till OD600 nm reach 0.4. Cells suspensions were centrifuged at 4 °C (7000 rpm, 5 min) to discard medium and sediment cells were washed twice with cold PBS buffer. Bacterial cells were resuspended in 1 mL of cold PBS buffer. Then 10 µM Fast-TRFS or Naph-EA-mal were incubated, respectively, with cell suspension in 96-well panel. The FI was measured immediately every 1 min over 15 min through VERSA microplate reader at 37 °C.

### 2.5. Detection of Thiol-Dependent Redox System in E. coli Mutants

Trx and Grx relevant mutants were used to compare the disulfide reducibility and total thiol level through Fast-TRFS and Naph-EA-mal probes. *E. coli* DHB4 mutants (*E. coli* wild type, *trxA*^−^ *grxA*^−^, *grxABC*^−^, *trxABC*^−^ or *gshA*^−^ mutants) were cultured overnight and then diluted 1:100 to grow till OD600 nm reach 0.4. Cell suspensions were centrifuged at 4 °C (7000 rpm, 5 min) to discard medium, and sediment cells were washed twice with cold PBS buffer. Bacterial cells were then resuspended in 1 mL cold PBS buffer. Cell suspensions were then incubated in 96-well panel with 10 µM Fast-TRFS or Naph-EA-mal, respectively. FI was measured every 1 min over 15 min using a VERSA microplate reader at 37 °C.

### 2.6. Statistical Analyses

Statistical analyses were carried out using Graph Pad Prism 7.0 (Graph Pad Software, La Jolla, CA, USA). In order to assess significance between *E. coli* wild type and other mutants, one-way ANOVA were used. *p* values of <0.05 were considered as significant difference. All assays were performed in triplicate.

## 3. Results

### 3.1. In Vitro Analysis of Disulfide Reducibility by Fast-TRFS

**Figure 1:**
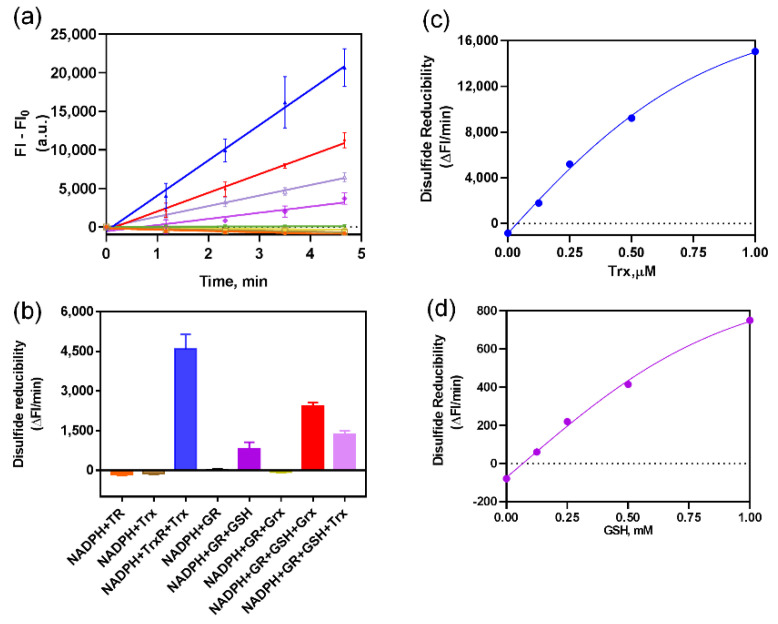
Fast-TRFS reactions with various thioredoxin enzymes and glutaredoxin enzymes in vitro. (**a**) Reduction of FAST-TRFS by various components in Trx and GSH-Grx systems. 10 µM Fast-TRFS was reacted with NADPH (200 µM), *E. coli* TrxR (50 nM) or/and *E. coli* Trx (1 µM); yeast GR (50 nM) or/and GSH (1 mM) or/and *E. coli* Grx (1 µM) in Tris (50 mM)/EDTA (1 mM) buffer (TE buffer, pH = 7.4). The compositions of the systems were labelled with same color as shown in (**b**). (**b**) Disulfide reducibility of various compositions of the Trx and GSH-Grx systems. Disulfide reducibility was represented by the slope of the liner curve of F-F_0_ versus time shown in (**a**). (**c**) Effects of Trx concentration on the reducibility of Trx system towards FAST-TRFS. 10 µM Fast-TRFS was incubated with NADPH (200 µM), TrxR (50 nM) and 0, 0.125, 0.25, 0.5 or 1 µM Trx. (**d**) Effects of GSH concentration on the reducibility of GSH/GR system towards FAST-TRFS. 10 µM Fast-TRFS was incubated with NADPH (200 µM), yeast GR (50 nM) and 0, 0.125, 0.25, 0.5 or 1 mM GSH.

Fast-TRFS is a specific and superfast fluorogenic probe previously used to test the activity of mammalian TrxR [37]. Fast-TRFS can react with mammalian TrxR within one step, which is the disulfide bond of the probe reduced by TrxR and then yielding blue fluorescence. However, bacteria TrxR contains an N-terminal active site while TrxR mammalian contains a C-terminal active site containing GCUG motif [38,39,40], which means TrxR of bacteria could not reduce Fast-TRFS. Thus, in order to assess the function of this probe in bacteria, partial or complete *E. coli* Trx or Grx system enzymes were incubated with Fast-TRFS in an in vitro 96-well panel. As shown in Figure 1a,b, Fast-TRFS was reduced by *E. coli* TrxR in combination with electron donor NADPH and Trx, which formed a complete donor–acceptor redox cycling system (Scheme 1b). Likewise, a significant increase in fluorescence intensity could be observed when GSH was coupled with NADPH and glutathione reductase (GR). NADPH or GSH alone, and NADPH/TrxR, NADPH/Trx, NADPH/GR and NADPH/GR/Grx did not possess the ability to reduce Fast-TRFS without the redox cycling (Figure 1a,b).

Furthermore, we used 10 mM DTT to reduce different concentrations of Fast-TRFS and detect the fluorescent intensity of reduced Fast-TRFS. There was a linear relation between the concentration of reduced Fast-TRFS and fluorescent intensity (Supplementary Figure S1). In addition, we investigated the effects of Trx and GSH concentration on the reduction of Fast-TRFS. Our results indicated that both of Trx and GSH could reduce Fast-TRFS in concentration-dependent manner with the complete system of NADPH/GR/GSH and NADPH/TrxR/Trx (Figure 1c,d). Curves of the reducibility versus enzyme concentration were well fitted by Michaelis-Menten equations. In vitro results also unveiled that Fast-TRFS was more efficiently reduced by the *E. coli* Trx system than the GSH-Grx system. Disulfide reducibility of the Trx system was 10-fold higher than that of the GSH system (Figure 1c,d). After converting the changes of FI into the changes of the concentration of disulfide in Fast-TRFS, the reaction rate of the reduction of disulfide in Fast-TRFS by Trx system could reach about 3.24 μM/min, which was at the similar level of the reaction rate of disulfide in insulin by the Trx system [31].

### 3.2. Detection of Fast-TRFS and Naph-EA-mal in E. coli Cells

**Figure 2:**
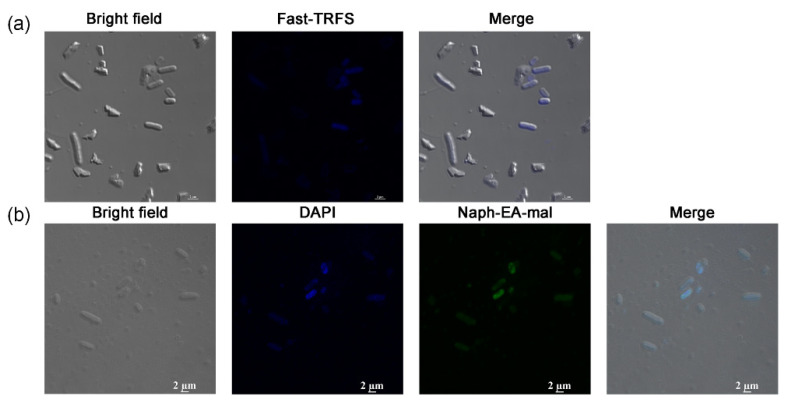
Images of Fast-TRFS and Naph-EA-mal in live *E. coli*. (**a**) Location of Fast-TRFS in *E. coli*. *E. coli* cells were treated with Fast-TRFS (10 µM) for 5 min, then bright field and fluorescence images were acquired. (**b**) Location of Naph-EA-mal in *E. coli*. *E. coli* were incubated with DAPI for 15 min and then stained with Naph-EA-mal (10 µM) for 15 min. The images of *E. coli* cells were captured using bright field, DAPI, green filter, respectively.

Above results demonstrated that Fast-TRFS is a substrate for *E. coli* thiol-dependent redox systems in vitro. This disulfide reduction occurred in a short period of time, which made real-time analysis of the disulfide reduction in bacteria become possible. However, whether this probe could work effectively in live *E. coli* cells to detect disulfide reduction was not clear. Therefore, a laser scanning confocal microscope was used to detect the ability of the probe to enter live *E. coli* cells. These results (Figure 2a) demonstrated that Fast-TRFS indeed permeable, as it was observed in the *E. coli* cytosol. This is consistent with previous observations that Fast-TRFS fluorescence could be detected in human HeLa cells in vivo [37]. However, bulk Fast-TRFS fluorescence, when compared at the same concentration, is not as strong as observed in mammalian cells [37]. Moreover, we used Naph-EA-mal to detect the total thiol level in these bacteria, which was used to detect LMW thiols including Cys, GSH and Hcy with high selectivity in vitro or in Hep G2 cells [41]. These results indicated that the two probes, Fast-TRFS and Naph-EA-mal, could enter the bacterial cells and they can be used in the analysis of cellular fast disulfide reducibility and total thiols.

### 3.3. Activity of Thiol-Dependent Redox System in Various Bacteria

**Figure 3:**
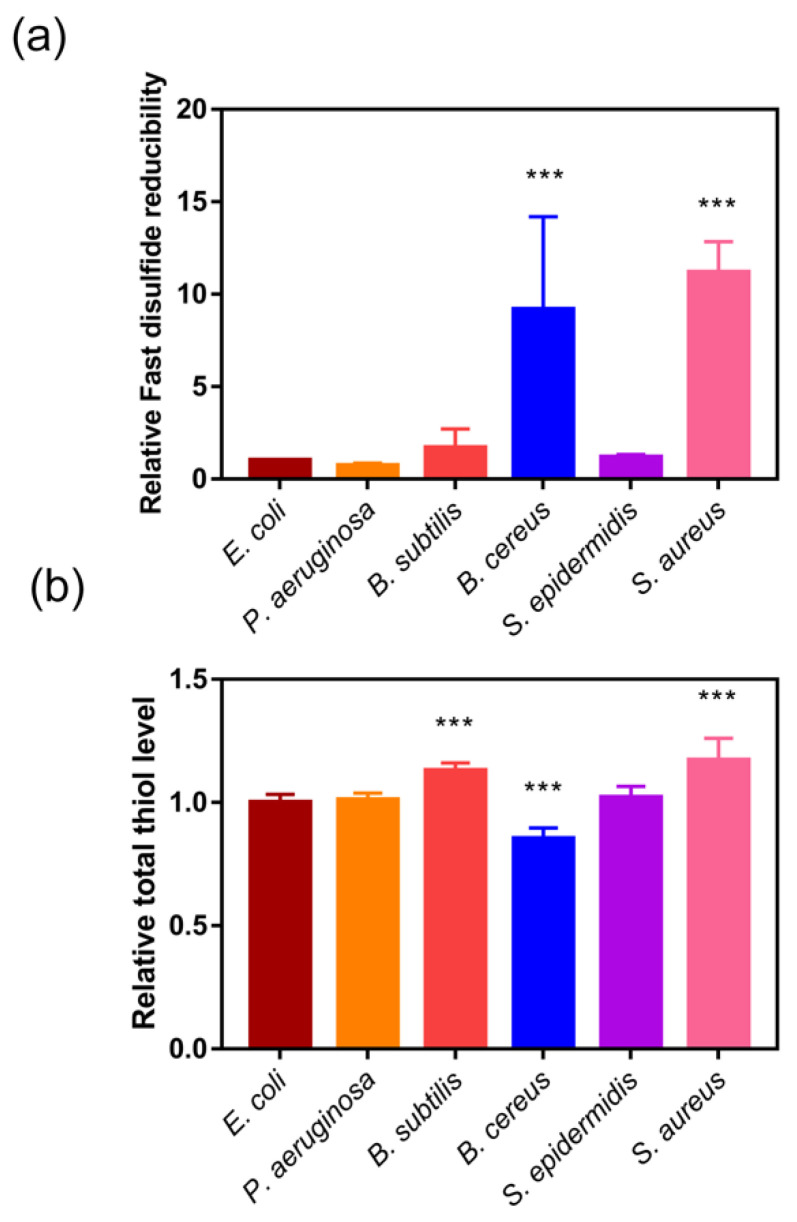
Fast disulfide reducibility and total thiol level in various bacteria. (**a**) Fast disulfide reducibility of bacteria detected by measuring the reduction of Fast-TRFS. *E. coli, P. aeruginosa, S. aureus, B. subtilis,* and *B. cereus* were incubated with Fast-TRFS (10 µM) at 37 °C to determine cellular disulfide reducing activity changes in every minute during 15 min. (**b**) Total thiol level of the bacteria detected by measuring the reduction of Naph-EA-mal. *E. coli, P. aeruginosa, S. aureus, B. subtilis*, and *B. cereus* were incubated with Naph-EA-mal (10 µM) at 37 °C to determine cellular thiol level changes every minute over 15 min. The reducibility was represented by the relative slope of curve of FI versus time. Data are shown as mean ± SD of three replicates. *** *p* < 0.001, One-way ANOVA.

Most Gram-negative bacteria have both Trx and Grx system, while most Gram-positive bacteria only have Trx system to maintain redox balance in cytoplasm [42]. Thus, we utilized Fast-TRFS and Naph-EA-mal to detect and compare the fast disulfide reducibility and total thiol level in *E. coli*, *P. aeruginosa*, *S. subtilis*, *S. cereus* and *S. aureus* (Figure 3). Interestingly, there was little difference in total thiol levels between Gram-negative and -positive strains (Figure 3b). Though Gram-positive bacteria *S. aureus* and *B. cereus* contained only Trx system [5,19,42,43], they had 10-fold higher disulfide reducibility compared to Gram-negative bacteria *E. coli* which was equipped with both Trx and GSH-Grx systems, indicating that a very active Trx system was present in these bacteria.

### 3.4. Contributions of Different Redox Genes in E. coli In Vivo

**Figure 4:**
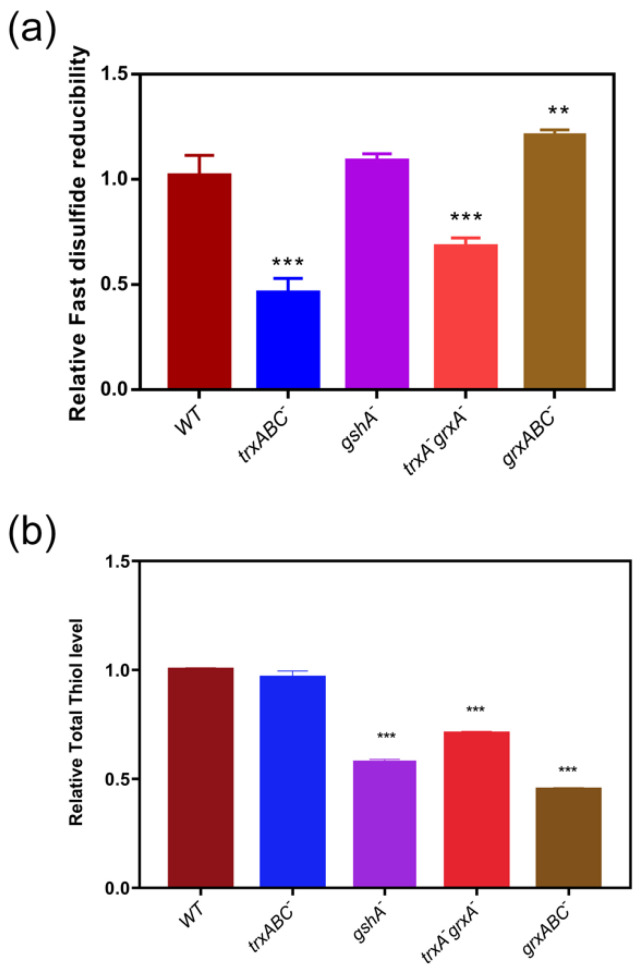
Fast disulfide reducibility and total thiol level in thiol-dependent redox gene null mutants of *E. coli*. (**a**) Fast disulfide reducibility in *E. coli* and its mutants. Fast-TRFS (10 µM) was incubated with *E. coli* strains including wild type (WT) and various *E. coli* mutants *grxABC*^−^ (Grx1, Grx2, and Grx3 deficient), *gshA*^−^ (Glutathione synthetase A deficient), *trxA*^−^*grxA*^−^ (Trx1 and Grx1 deficient) and *trxABC*^−^ (Trx1, TrxR and Trx2 deficient) at 37 °C to determine cellular disulfide reducing activity changes in every minute during 15 min. (**b**) Total thiol level in *E. coli* and its mutants. Naph-EA-mal (10 µM) was incubated with *E. coli* strains including WT, *grxABC*^−^, *gshA*^−^, *trxA*^−^*grxA*^−^ and *trxABC*^−^ individually at 37 °C to determine cellular thiol level changes every minute over 15 min. Then the fluorescence intensity in various mutant *E. coli* was measured. Reducibility was represented by the relative slope of the FI curve versus time. Data are shown as mean ± SD of three replicates. ** *p* < 0.01, *** *p* < 0.001, One-way ANOVA.

To verify the contributions of the disulfide reductases in in vivo disulfide/dithiol redox regulation, in particular to confirm whether thioredoxin is the predominant system to reduce Fast-TRFS in bacteria, we examined the reducibility of disulfides and LMW thiols level by Fast-TFS and Naph-EA-mal in *E. coli* strains including *E. coli* wild type, *trxABC*^−^, *grxABC*^−^, *trxA*^−^, *grxA*^−^ and *gshA*^−^. As shown in Figure 4a, lack of Trx system (*trxABC*^−^) or Trx1 and Grx1 (*trxA*^−^*grxA*^−^) resulted in loss of almost half of disulfide reducibility loss, while *gshA* deficient strain had no significant effects on the activity of disulfide reducibility. The Grx deficient strain (*grxABC*^−^) showed even a slight increase in reducibility of disulfide compared to *E. coli* wild type. In contrast, total thiol level in the *trxABC*^−^strain was not significantly different when compared to wild type; while *gshA*^−^, *trxA*^−^*grxA*^−^and *grxABC*^−^ strains had lower thiol levels compared to wild-type *E. coli.* These results confirmed that fast disulfide reducibility of bacteria was determined by the Trx systems, rather than the total thiol level or Grxs.

## 4. Discussion

The in vivo reactions catalyzed by thiol-dependent antioxidant enzymes are not easily monitored in real time due to the fast but overlapping properties of the enzymatic reaction. Most of the frequently used methods designed to analyze Trx and Grx activities only work in vitro for tissue or cell extracts [34,44]. Fast-TRFS is a specific and superfast fluorescent probe that has been designed for measuring mammalian thioredoxin reductase (TrxR) [37]. Mammalian TrxR enzymes are a family of selenoproteins with a selenocysteine (Sec) residue at their C-terminal redox center [38]; while TrxR in *E. coli* is a family of flavoenzymes that harbor an active-site dithiol-disulfide [39,40]. Here, we used Fast-TRFS to detect the fast disulfide reduction capacity of bacteria. Similar to another mammalian TrxR probe, TRFS-green, which was used to detect bacterial Trx and Grx [36], Fast-TRFS was not a substrate of bacterial TrxR plus NADPH, and NADPH, but a substrate of both complete Trx and GSH-Grx systems in vitro. Though the excitation wavelength of Fast-TRFS was close to the absorbance wavelength of NADPH, the presence of NADPH caused the increase of fluorescent intensity in the beginning point, and a little quenching in fluorescence (Figure 1), but did not contribute to subsequent fluorescent intensity increase due to disulfide cleavage.

Different with TRFS-Green, Fast-TRFS was also a substrate for NADPH/GR/GSH, with a lower efficiency compared to Trx system. Nevertheless, Fast-TRFS could be used to represent the fast disulfide reduction, which lasted 1–5 min. This makes the real-time monitoring in bacteria feasible. Very surprisingly, in vivo experiment to with redox gene null *E. coli* strains and various bacteria unveiled that *B. cereus* and *S. aureus*, which lack the GSH- Grx system, contain 10-fold higher fast disulfide reducibility towards Fast-TRFS. This is consistent with the fact that Trx system deficiency resulted in significant decrease in fast disulfide reduction, but not total thiol levels detected by Naph-EA-mal (Figure 4). This is completely different with the results detected by TRFS-green, which show that GSH-Grx, especially Grx2 and Grx3 are the predominant disulfide reductases in *E. coli*, corresponding to expression level of the redoxins in the bacteria. Moreover, disulfide reductase activities detected by TRFS-green in *B. cereus* and *S. aureus* etc. are much lower than that in *E. coli* [36].

The disulfide reduction reaction catalyzed by Trx via a dithiol exchange mechanism, while reduction catalyzed by Grx proceeds via dithiol or monothiol mechanism. However, from the view of reaction kinetics, Trx and Grx may belong to two different types of disulfide reductases. Trx is responsible for the fast disulfide reduction, whereas Grx2&3 are responsible for the slow disulfide reduction. This may be a reasonable explanation for the difference obtained from the probes. The high efficiency of Fast-TRFS reduction by the Trx system indicated that the Trx system is a predominant player in the fast disulfide reduction rates that keep the substrate enzymes active.

The disulfide reduction rate is an important factor for cellular function. In *B. subtilis*, evidence suggests that the redox flux of the Trx system modulates the rate of sulfide production in cysteine desulfurase and the activity of the Trx system also depends on the rate of disulfide formation [45]. Furthermore, evidence from crystal structure analysis indicates that only one out of two binding sites of the *B. cereus* TrxR homodimer is occupied with NADPH, indicating a possible asymmetric co-substrate binding in TrxR [46]. In this study, *B. cereus* showed much higher disulfide reducibility among *E. coli*, *P. aeruginosa* and *B. subtilis*. Besides, a previous study identified two thioredoxin-like proteins TrxP and TrxQ, which could reduce protein disulfide as a potential regulatory mechanism [47]. This may account for the highest disulfide reducibility of *S. aureus* among other strains.

The thiol-dependent redox regulation controlled by Trx and Grx systems plays a critical role in a wide range of cellular activities from antioxidant function, redox signaling transduction, oxidative protein repair to DNA synthesis and repair. In particular, GSH-Grx is lacking, which makes the Trx system is essential for some pathogenic bacteria such as *S. aureus*, *Helicobacter pylori*, *Mycobacterium tuberculosis* [6,8,48,49], and they can be potential drug targets [5,44,49,50]. These properties make real-time detection of the cell redox state an attractive topic for research and drug discovery. The results shown here that thioredoxin system activity detected by Fast-TRFS in *S. aureus* is much higher than *E coli*, and *P. aeruginosa,* and other bacteria, imply the significance of the Trx system in *S. aureus*. Thus, Fast-TRFS should be a very useful probe to detect the redox state change under various oxidative stress in such bacteria.

## 5. Conclusions

In this study, we have shown that two probes, Fast-TRFS and Naph-EA-mal, can be used in the real-time analysis of the redox state in bacteria. In particular, the Fast-TRFS probe can be a convenient tool to detect the activity of the disulfide reductase system. In addition, Naph-EA-mal was also another good probe to detect levels of thiols in bacteria. Using these probes, we revealed that the fast disulfide reduction reactions in vivo are controlled by the thioredoxin system and not determined by total thiol levels or the Grx system. The approach based on the Fast-TRFS is very sensitive and fast, this also resulted in a drawback of the approach that the experiment needs to be performed very quickly to obtain the initial reaction rate to represent the disulfide reductase activity. Moreover, the presence of NADPH, etc., seems to result in a little quenching of fluorescence. Thus, measurement requires to finish in a short time to avoid this disturbance. The delay of the measurement may result in the variance of absolute value. The other limitation is that the probe only detects the disulfide reduction with a fast reaction rate. To have an overview of the cellular redox state change, the other fluorescent probes such as TRFS-green, Naph-EA-mal and so on are needed to detect various specific reactions.

## Data Availability

All of the data is contained within the article and the supplementary materials.

## Supplementary Materials

The following supporting information can be downloaded at: https://www.mdpi.com/article/10.3390/antiox11020377/s1, Figure S1: Fluorescent intensity of Fast-TRFS reduced by DTT.
