# Supplementary material for: A Fluorescent Probe to Detect Quick Disulfide Reductase Activity in Bacteria
Source: Antioxidants (Basel). 2022 Feb 13;11(2):377. doi: 10.3390/antiox11020377 (PMC8868778; doi:10.3390/antiox11020377)
Supplement: Supplementary file 1 [file antioxidants-11-00377-s001.zip › antioxidants-1572411-supplementary.pdf]

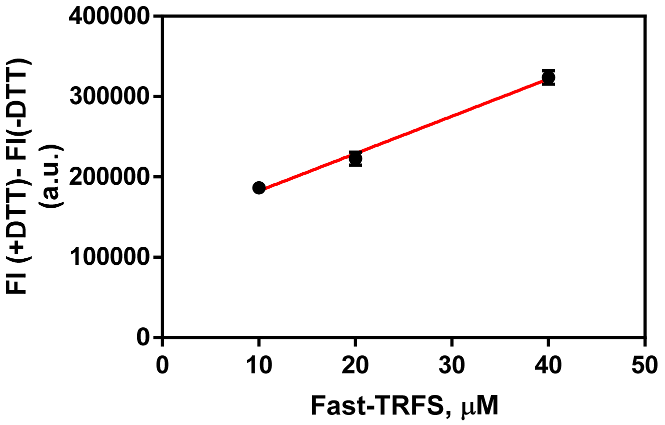

Supplementary Figure S1. Fluorescent intensity of Fast-TRFS reduced by DTT. Fast-TRFS (10, 20, 40 μM) was reacted with DTT (10 mM) at 37 °C in 20 min. FI was measured with fluorescent plate reader. The assays were performed in triplicate.
